# Biological and ergonomic risks of radiology technicians in a conventional radiology service

**DOI:** 10.47626/1679-4435-2021-617

**Published:** 2021-12-30

**Authors:** Tiago Jorge Anderson, Francine Lima Gelbcke

**Affiliations:** 1 Hospital Universitário Professor Polydoro Ernani de São Thiago, Universidade Federal de Santa Catarina, Florianópolis, SC, Brazil.

**Keywords:** occupational health, occupational risks, hospital radiology department, working conditions, saúde do trabalhador, riscos ocupacionais, serviço hospitalar de radiologia, condições de trabalho

## Abstract

**Introduction:**

Radiology technicians are exposed to occupational risks in their daily work. Among these, there are biological and ergonomic risks.

**Objectives:**

To describe the biological and ergonomic risks in the work process of radiology technicians in a conventional radiology service.

**Methods:**

This was a qualitative, descriptive, and exploratory research. Data collection was conducted through semi-structured interviews with 12 radiology technicians, participant observation, and consensual validation with nine workers. Content analysis was based on the assumptions of the thematic analysis.

**Results:**

Exposure to body fluids, contamination of work instruments by biological agents, no disinfection, and no use of personal protective equipment were found to be biological risks. Excessive weight manipulation, inappropriate physical structure, night work, and work instruments that do conform to the principles of body mechanics were identified as ergonomic risks.

**Conclusions:**

The mentioned occupational risks are related to the work conditions of the health service and to inadequate body positioning of radiology technicians.

## Resumo

**Introdução:**

Os profissionais de radiologia estão expostos a riscos ocupacionais no seu cotidiano profissional. Entre eles, temos os biológicos e os ergonômicos.

**Objetivos:**

Descrever os riscos biológicos e ergonômicos presentes no processo de trabalho dos profissionais de radiologia em um serviço de radiologia convencional.

**Métodos:**

Pesquisa qualitativa, descritiva e exploratória. Utilizou-se como técnica de coleta de dados entrevista semiestruturada com 12 profissionais de radiologia, observação participante e validação consensual com nove trabalhadores. A análise do conteúdo embasou-se nos pressupostos da análise temática.

**Resultados:**

Exposição a fluidos corporais, contaminação de instrumentos de trabalho por agentes biológicos, não realização de desinfecção e não uso de equipamentos de proteção individual foram evidenciados como riscos biológicos. Manipulação excessiva de peso, planta física inadequada, trabalho noturno e instrumentos de trabalho que não respeitam os princípios da mecânica corporal foram identificados como riscos ergonômicos.

**Conclusões:**

Os riscos ocupacionais evidenciados estão relacionados às condições de trabalho do serviço de saúde e a posturas inadequadas dos profissionais de radiologia.

## INTRODUCTION

The work process in health consists of different professions, involving workers with mastery of different expertise and procedures and encompassing multiple types of knowledge and procedures, in order to assist individuals in health situation.^1^ One of these professionals are radiology technicians.

Radiology technicians are health care workers that perform tests in the radiology field, i.e., they act in the production of images from within the body that allow diagnosing disease situations. Their main functions involve planning, performing, and assessing all the radiology techniques used in health diagnosis, prevention, and promotion, resorting, for this purpose, to technologically advanced equipment.

It is a fact that, in this work process, radiology technicians are exposed to several elements that may interact with worker’s body and cause strains, known as occupational risks. Among these elements, we highlight ionizing radiation, presence of micro-organisms in body fluids and secretions, accelerated work pace, scarcity of human resources, and excessive weight manipulation.^2^

Occupational risks are characterized as elements present in the work process of a professional category. By dynamically interacting between themselves and with workers, these risks may be responsible for generating new adaptation processes and, consequently, occupational burnout, which is defined as loss of body and psychic capacity.^3^ Previous identification of the occupational risks present in a work process may subsidize the implementation of actions of workers’ health promotion, protection, and prevention.^4^ Therefore, it is necessary to recognize these risks in specific work processes.

In the context of radiology techniques, an integrative review addressing the occupational risks of health care professionals identified the following ergonomic risks: physical effort related to standing position, travelling long distances, handling and lifting heavy objects, poor postures, and night shifts.^5^ With regard to biological risks, direct contact with patient’s fluids and secretions; manipulation of contaminated material during the processes of disposal, transportation, and cleaning of materials; and use of objects in patient care were mentioned.^6^

In view of the need of previously recognizing to which occupational risks these professionals are exposed, we designed the following objective: to describe the biological and ergonomic risks present in the work process of radiology technicians in a conventional radiology service.

## METHODS

The present article derives from the master’s thesis entitled “Workload and burnout of radiology technicians in a conventional radiology service.”^7^ This is a qualitative, exploratory, descriptive research that used the Italian Worker Model (IWM) as a methodological reference in the stage of consensual validation.

The use of qualitative research allows for understanding complex phenomena within a context to be investigated, establishing interfaces between concepts, representations, beliefs, and behaviors and respecting intersubjectivity.^8^ Meanwhile, the present research aimed to understand the phenomenon of occupational risks in the radiology unit of a public hospital located in southern Brazil. For this purpose, observation was used as a methodological step. In order to value the subjectivity and intersubjectivity of radiology professionals, individual interviews and consensual validation of the IWM were carried out.

The research was conducted at a general hospital linked to State Health Secretariat of Santa Catarina that provides medium and high complexity care at outpatient and hospital levels. This institution was chosen because it is a reference in orthopedics and trauma in the state and the second specialty with the greatest number of inpatient beds. This aspect is highlighted due to the existing interface between the diagnosis and imaging service and the specialty of orthopedics and trauma. This interconnection contributes to the comprehensive discussion of the investigated phenomenon in all its dimensions, considering the constant execution of conventional radiology tests.

Inclusion criterion for this research was radiology technicians who worked in conventional radiology. Of the initial population of 19 radiology technicians, five were excluded because they worked in the computed tomography unit. Of the remaining 14, two were not included in the sample because they were on leave of absence for medical treatment. Therefore, the research sample consisted of 12 technicians. For data collection, semi-structured interview, participant observation, and consensual validation were employed.

The first stage consisted of the interview with the 12 technicians included in the study. The questions addressed the occupational risks in the work process of the technicians interviewed. Interviews were recorded and then transcribed. The second stage consisted of participant observation, following a previously designed script. Reflexive and descriptive notes were written in a field diary and then transcribed. There was a total of 50 hours of observation. It is worth emphasizing that the three work shifts were observed.

After these stages, pre-analysis of the data was made based on the framework about occupational risks. The previously analyzed data underwent the stage of consensual validation of IWM, defined as the process through which the recorded data are consensually validated by a homogenous work group.^9^ With the use of this methodological model, it was possible for workers to achieve a leading role. This referential framework advocates that demands should emerge from workers rather than from experts’ analysis of health experts and establishes that no expert knowledge external to the work group may define the occupational risks to which workers are exposed. It applies the concept of homogeneous group, i.e., a group of workers who share common work conditions, and the concept of consensual validation, in which the homogeneous group deliberates on the occupational risks present in the work environment and formula proposals to improve work conditions.^10^

Thus, workers’ leading role was ensured in the research, since pre-analyzed data were subjected to discussion by the homogeneous groups, and a risk was considered occupational only if it was recognized as such by the homogeneous groups. Three groups were formed, with three professionals each. Three professionals were not interested in participating in this stage. The discussion with the homogeneous group was recorded and transcribed.

Data were analyzed through thematic analysis. This method allows for identifying, analyzing, and reporting emerging themes, also resulting in the interpretation of the several aspects related to the research theme.^11^ Occupational risks present in the work process of radiology technicians emerged as a theme, with biological and ergonomic risks being the reference for the present article.

This research was submitted to Plataforma Brasil and was approved under opinion number 1.020.563. Participants received an informed consent form. In order to ensure participants’ anonymity, alphanumerical codes were used, consisting of the letter I (interviewee) and a cardinal number in ascending order (1, 2, 3, ...). Consensual validations were also alphanumerically coded, using the letter G (group) together with a cardinal number (1, 2, 3) according to the sequence of the interviews.

## RESULTS

Of the total of radiology technicians interviewed, 58.3% were female and 41.7%, were male. With regard to age, 8.3% were between 20 and 30 years old; 50%, between 30 and 40 years old; 33.3%, between 40 to 50 years old; and 8.4%, between 50 and 60 years old. With regard to schooling, 66.7% had a high school degree, while 33.3% had a higher education degree. In relation to working time, 16.7% had from 0 to 10 years; 75%, from 10 to 20 years; and 8.3%, from 20 to 30 years. It was found that 91.7% work overtime, whereas 8.3% do not. Moreover, 33.3% had a double employment relationship, and 66.7% do not. With regard to the work shift, 50% worked in the morning period, and 50% worked in the afternoon period.

In the work process of radiology technicians, it was observed that, during care to trauma patients, there is exposure to body fluids, such as blood. The discourse of one of the professionals corroborates the observed data:

Like what happened today, at the polytrauma unit, the patient arrived with blood, we had to handle him with gloves, then you are contaminating the equipment, and after that you take the equipment without gloves! (I1)

The discourse also evidences the contamination of work instruments due to manipulation of equipment after contact with patients. Constant disinfection of radiographic cassettes was not observed in the provision of patient care, whether tests were performed at bedside or in the radiology and imaging unit.

When tests were performed at the bedside of patients with multi-resistant bacteria, radiographic cassettes were wrapped in plastic for protection. However, it was observed that this plastic was not removed after the test was completed, and radiographic cassettes returned to the mobile device with the contaminated plastic. This factor was mentioned to the group during consensual validation, which stated that “some employees take the radiographic chassis with the plastic and return it with the plastic” (G2).

In consensual validation, a debate emerged about the lack of availability of personal protective equipment (PPE) by the institution:

Except when there’s not! The test has to be done! You arrive at the ICU and see that there is no [impermeable] apron in the ICU... The test had to be done, I did it! (G2)

What happened somewhere and I found it absurd was... arriving at the semi-intensive care unit and having a single apron to handle all patients because there was shortage of aprons in the hospital. (G2)

As an aggravating factor, a lack of awareness on the type of required precaution during patient care was also found. Although standard precaution was taken, some discourses showed insecurity with regard to how vulnerable these professionals may be due to the lack of indication on the type of precaution to which the patient should be subjected. This somehow increases the level of exposure to biological loads:

You arrive there...all right, when the patient is on isolation you known! But when he or she is not? Ah, I forgot to put the little card... Well, now it’s too late! In hospitals it’s basically the way it is, you know.. Time will what you got. (I11)

I think that what happens is that we go to the ICU or go to another unit, they assume that you’ve already know you have that... The other day we found a patient there in the street and saw that it was an ICU patient, he was KPC positive, but nobody told us that, we transferred the patient, handled him, did everything... At the end they commented that the patient was diagnosed as KPC positive. (G2)

Consensual validation evidenced the risk of vulnerability to contamination by pathogen microorganisms associated with direct patient care and to the lack of indication on the type of precaution to be taken during this care:

We end up assisting everybody who arrives at the emergency room and, as you know, there’s no indication on precaution and we end up learning later that the patient had tuberculosis, or meningitis, as already happened to me when a patient had to be medicated because he was confirmed with meningitis. (G1)

During the work process, lack of standard precaution was observed in all care actions. Firstly, professionals did not wear gloves during some tests. Furthermore, hand hygiene was rarely performed; the examination table was disinfected only when there was visible exposure of blood; and the radiographic mobile device was not decontaminated between tests in different patients, even when examination was performed at the bedside of patients with indication of contact precaution. A behavior of adaptation was also observed in face of the constant exposure to biological loads in the everyday care practices:

[...] I believe that we relax a little regarding this because it’s too common. So, we don’t take some precautions that we’re supposed to because it’s routine. We end up losing sight of the danger! Every day the danger is very close, so close that it becomes normal. (I7)

With regard to ergonomic risks, excessive weight manipulation could be observed when transferring of patients from stretcher to examination table, carrying radiographic cassettes, positioning body structures of dependent and semi-dependent patients, and performing bedside tests. In the stage of patient transfer from stretcher to examination table, especially in the night shift, with an insufficient number of professionals to perform the transfer.

Another factor that emerged in the discourse of radiology technicians as ergonomic risk is related to work instruments that do not comply with the principles of body mechanics. It was found that some transportation stretchers did not have height adjustment, and their higher than the examination table, which hampers patient mobilization and transfer. The locks of transportation stretchers did not work, and their wheels did not spin. It is also worth mentioning that examination tables did not have height adjustment:

We don’t have equipment, there is no table, which is compatible with the stretcher used by firefighters, and sometimes we have to climb on the table. (I7)

To perform patient positioning, radiology technicians need to lift patient’s chest with one hand and positioning the radiographic cassette with the other. Most bedside tests are chest ones and are performed in patients without clinical conditions to be referred to the radiology unit. All these factors lead to excessive weight manipulation and may result in inappropriate body positioning, which may cause musculoskeletal disorders.

While performing tests in multiple trauma patients, there is the need to use a great number of radiographic cassettes, due to high number of structures and incidences to be obtained. Since there is no specific equipment to perform the transfer between the room where cassettes are stored and the room where images are obtained, the transportation of cassettes is made by radiology technicians. There was a situation in which the professional carried eight 35 × 43 radiographic chassis to perform an x-ray test in a patient with multiple trauma. Excessive weight manipulation in the daily work of radiology technicians is evidenced in the following discourse:

The weight of cassettes, which sometime is a great number of tests from the same patient and we have to carry many cassettes, or patients come in a wheel chair and we have to help them to get out of it, a certain physical strength, depending on patient’s weight, or on a strength, we have to transfer the patient to an examination table, sometimes we don’t have people enough to do this kind of work, which is heavy. (E3)

Participants also highlighted that they had to perform repetitive stress activities to handle the work instruments:

This exchange of cassettes when we are taking the image, each image acquisition, or even when carrying or handling the ampoule, the examination stand, which gets stuck, the equipment is always heavy because it’s not well calibrated, sometimes screws become loose, as the maintenance staff say, and the rail becomes really heavy, for us to handle it. (I5)

In this part we always make a lot of effort, which is changing films, changing cassettes, this repetitive stress of opening the bucky, placing them, which are always very heavy. (I5)

Another ergonomic risk present in the work of these professionals is related to night work. The conventional radiology service works 24 hours a day. In the night period, two professionals work per shift, who both provide care in the unit and perform bedside tests in hospital wards. In professionals’ discourse, the lack of an exclusive work team for the night shift also emerged, which contributes even more to evidence the night shift as a physiological load:

I think our working hours, this mixed shift, day and night! [...] You work in a 5-hour day shift and then in a 12-hour night shift. For me, I feel it’s extremely stressful. (I7)

The physical structure of the unit was also noted as an element that may cause occupational burnout. The radiology and imaging unit has three fixed x-ray devices and an image digitization room. One of the rooms is located in the corridor opposite to the digitization room, which implies long travels to perform the requested tests. As previously described, there is the need to transport x-ray cassettes. It is revealed that the association of inappropriate physical structure associated with the need of weight manipulation and long travels leads workers to expose themselves to inadequate working conditions that characterize ergonomic risk.

## DISCUSSION

Blood is considered the organic fluid with which health care professionals have most contact in accidents with biological material. Inappropriate body positioning of professionals contributes to the high exposure to this risk.^12,13^ The use of PPE to prevent exposure to fluids and secretions is neglected by the majority of professionals during provision of direct patient care.^14^

Patient diagnosis is considered a favorable factor to greater adherence to the use of preventive measures. Conversely, the awareness of a negative diagnosis for infectious and contagious diseases may contribute to non-adherence to measures of standard precaution. Health care professionals may adopt these measures in their work routine, in order to prevent occupational contaminations and patients’ exposure to infections related to health care.^15^ It is a fact that, although standard precaution measures are internationally recommended, they are partially adopted by health care professionals. There is a need for permanent education in order to achieve greater adherence to these measures.^16^

It is worth highlighting that factors such as exposure to emotional stress, work overload, long working hours, and double employment relationship may contribute to non- adherence to the standard health care precautions.^13^ As previously shown, 91.7% of professionals worked overtime, and 66.7% had a double employment relationship, which may contribute to exposure to occupational risks.

With regard to the contamination of x-ray cassettes, a study conducted in a teaching hospital in Brazil found the presence of pathogens after the conduction of radiographs and that disinfection of cassettes is not performed during care of patients who require x-ray tests.^17^ Another study pointed that, in a sample of 67 radiology technicians, 91% did not decontaminate the material after the tests, and 100% did not perform hand hygiene with the proper technique. Furthermore, it was found that 91.42% of radiographic cassettes used in bedside tests contained microorganisms.^18^

The aforementioned data were also observed in the unit analyzed in the present study, revealing a worrying reality in term of risk of transmission of pathogen microorganisms during the conduction of radiographic tests. There is the need to implement educational measures that guide these professionals on the adoption of the practice of decontaminating radiographic chassis during patient care, as well as the implementation of care protocols addressing this theme.

The use of barriers methods between the radiographic cassette and the patient is recommended to prevent contamination. Meanwhile, it is suggested to cover the chassis with a sterile and disposable bag during bedside tests.^18^ The use of a plastic bag was observed in the work process of radiology technicians in the present research; however, the bag was not removed after the test was completed, which may contribute to the propagation of pathogen microorganisms.

The absence of PPE is a reality experienced by health care professionals. This situation could be observed with greater evidence in the emergency caused by the coronavirus disease (covid-19) pandemic.^19^ It is important to emphasize that the Brazilian legislation sets forth that PPE should be available in a sufficient amount in workplaces, so as to ensure replacement or immediate provision.^20^

In a study with radiology professionals, 56% of the total of 29 workers reported that the institution sometimes provides or does not provide PPE to workers.^21^ Moreover, half of the respondents did not find the use of PPE important, pointing to urgency of care, great patient demand, and difficulty of PPE availability as factors that contribute to the non-use of PPE.^21^ In some scenarios, lack of PPE was observed. However, it is worth highlighting that, in other situations, adherence to the use of PPE is low by a portion of professionals.

With regard to ergonomic risks, studies indicate that carrying, lifting, or moving heavy materials and equipment; working with the upper limbs above the head or away from the body; inadequate furniture; moving patients in bed; transferring patients; and moving or removing patients with a number of professionals below the desirable and with inappropriate equipment are considered as occupational risks in the context of work in health.^22^ Factors in the work environment of radiology technicians that may lead to burnout included working with old and scrapped instruments, lack of preventive and corrective maintenance of equipment, inappropriate infrastructure in the work environment; and carrying radiographic cassettes for obtaining images,^23^ which were also reported by workers in the present study.

In relation to night work, it is knowingly associated with increased risk of detrimental health effects, including disruption of circadian rhythm, disturbed sleep, and suppression of melatonin levels. These factors may contribute to disturbances in several physiological and behavioral processes, causing workers to become ill. Night work is considered a risk factor for acute myocardial infarction, visceral obesity, dyslipidemia, abnormal blood pressure, changes in blood glucose levels, peptic ulcer, depression, and insomnia.^24^

## CONCLUSIONS

The following biological risks were identified: exposure to body fluids (especially blood); contamination of work instruments with biological agents; no implementation of standard precautions when performing radiographic tests; no disinfection of work instruments and surfaces; and no use of PPE, whether due to worker’s negligence or to lack of availability in the institution. As occupation risks, excessive weight manipulation, whether when positioning patients or manipulating work instruments; inappropriate physical structure; night work; and work instruments that do not conform to the principles of body mechanics.

Occupational risks are mostly related to inappropriate work conditions, such as scarcity of human and material resources, which are so common in the work scenario in health care. The present study also revealed that, in some situations, workers do not take the measures that prevent exposure to occupational risks, i.e., they do not follow standard precaution measures and do not wear PPE.

The use of consensual validation should be highlighted as a way to value workers’ subjectivity and intersubjectivity, a desirable factor in qualitative research. The main limitation of the study is the fact that only one work reality was observed. It is necessary to expand the number of work scenarios in order to achieve a deeper understanding on the occupational risks present in the routine of radiology technicians in conventional radiology.
